# Some Peculiarities of Anthrax Epidemiology in Herbivorous and Carnivorous Animals

**DOI:** 10.3390/life12060870

**Published:** 2022-06-10

**Authors:** Irina Bakhteeva, Vitalii Timofeev

**Affiliations:** State Research Center for Applied Microbiology and Biotechnology (SRCAMB), 142279 Obolensk, Russia; bahtejeva@mail.ru

**Keywords:** *Bacillus anthracis*, anthrax, herbivores, carnivores, epidemiology

## Abstract

Anthrax is an especially dangerous zooanthroponosis caused by the Gram-positive spore-forming bacterium *Bacillus anthracis*. A notable feature of this disease is the difference in susceptibility to it among different groups of animals. Anthrax primarily affects herbivorous ungulate mammals; they are easily infected, and their disease often leads to rapid, even sudden, death. However, predators and scavengers are extremely resistant to anthrax, and if they become infected, they usually become mildly ill. As the result of the increased sensitivity of ungulates to anthrax and the possibility of disease transmission from them to humans, most studies of anthrax have focused on the diagnosis, prevention, and treatment of infection in farm animals and humans. The issues of anthrax in other animals, such as predators, and the peculiarities of anthrax epidemiology in wild ungulates have not been sufficiently detailed in the literature. In this article, we provide a review of literature sources that describe the differential susceptibility to infection of various groups of animals to anthrax and some epidemiological features of anthrax in animals that are not the main hosts of *B. anthracis*.

## 1. Introduction

Anthrax is an especially dangerous disease caused by the Gram-positive spore-forming bacterium *Bacillus anthracis*. *B. anthracis*, a typical member of the genus Bacillus, is capable of forming endospores outside the host organism that are extremely resistant in the environment [1,2].

In the overwhelming majority of cases, it is by spores that macro-organisms are infected. Spores entering the body of a warm-blooded host germinate and turn into vegetative cells that multiply at the penetration point or in the nearest lymphatic tissues. Then, as the disease progresses, *B. anthracis* cells spread throughout the body by the lymphohematogenous route and cause hemorrhagic necrotic lesions, toxemia, and sepsis, which can lead to death. Depending on the route of entry of spores into the body, infection can take different clinical forms. When spores enter skin lesions, such as cuts, abrasions, and microcracks, a skin form of anthrax develops, which manifests as local necrotic skin lesions and local edema, which disappear over time [3]. In some cases, however, the disease can progress to the generalized stage and become fatal [4]. The inhalation of an aerosol containing spores can cause pulmonary anthrax, which is nearly always fatal if left untreated. It manifests as a flu-like illness with increasing severity of symptoms, up to edema of the chest organs, bacteremia, and even brain damage, so-called anthrax meningitis [3,5]. Oropharyngeal and gastrointestinal anthrax occur with the consumption of food contaminated with *B. anthracis* spores. These forms are manifested by oral ulcers, lymphadenopathy of the cervical and/or submandibular lymph nodes and neck edema in the case of the oropharyngeal form, or ulcerative lesions and edema of the stomach and intestines in the case of the gastrointestinal form. Both forms of the disease can lead to severe intoxication, bacteremia, and death [3,6].

Gastrointestinal anthrax has the greatest epidemic significance in nature. Consumption of other organisms is the simplest and most obvious means of macro-organism interaction in natural conditions. The anthrax microbe is perfectly adapted to such relationships between macro-organisms, making them the way of its circulation in natural ecosystems. When eating near-ground plants, herbivores consume some amounts of soil, which can be contaminated with *B. anthracis* spores and, accordingly, be a source of anthrax infection. In this case, gastrointestinal anthrax develops, from which the sick animal quickly dies. Within the organism of a living host, *B. anthracis* cells are able to exist and multiply but not to form endospores. After the death of the host, the vegetative cells of the anthrax microbe die during the decomposition of the corpse under the influence of putrefactive microorganisms. Therefore, in order for the life cycle of the anthrax microbe to be complete, some of its cells must travel from the organism of the infected host into the environment, where they form spores again. In part, this process occurs through the bloody discharge from a sick animal at the terminal stages of infection, along with infected blood, a certain number of *B. anthracis* cells are released to the environment. Predators and scavengers make a more significant contribution. They eat sick herbivores (which are weakened by the disease and are easy prey), and the corpses of animals that have died from anthrax. Since anthrax mainly affects large animals, in most cases, predators and scavengers cannot swallow such prey whole, and they are forced to tear the eaten body into pieces. In this case, blood and tissue scraps contaminated with *B. anthracis* inevitably enter the soil, where the process of endospore formation takes place. The most intriguing aspect is that the predators and scavengers, themselves, after eating an animal that was sick with anthrax, almost never become infected, and if they do, they develop a mild form of illness. Thus, instead of becoming another victim of infection, they mainly act as anthrax disseminators.

Thus, in natural ecosystems, anthrax exists as a food infection of herbivores that circulates because of stable trophic chains, “plants—herbivores—predators”, and because of the differential susceptibility to infection of these groups of animals.

Only humans avoid this scheme because (1) their chances of being eaten by predators during illness, and even after death, became vanishingly small in the early stages of the development of society; (2) there are religious, cultural, and, later, sanitary and regulatory prohibitions on eating animals that have died from the disease (not always strictly enforced in areas lacking food security or significant food inspection, especially during times of famine, see for example [7]); (3) for humans, contact and inhalation routes of anthrax infection are more significant than the trophic route typical of natural conditions. During the processing of livestock raw materials contaminated with *B. anthracis* spores for the production of clothing, tools, jewelry, housing construction, etc., there is the possibility of spores getting into skin lesions, as well as the formation and subsequent inhalation of spore-containing aerosols. After the lethal potential of such aerosols was studied, the idea arose that such aerosols could be created artificially and used for military purposes to deliberately infect people. As a result, the most common cutaneous anthrax and the most dangerous inhalation anthrax have acquired the greatest significance for humans. It is on these forms of the disease that most research is focused. As for non-human anthrax, the scientific literature mainly presents the results of anthrax studies in farm animals, which is quite natural, considering the role of domesticated ungulates in human civilization. Both human and cattle forms of anthrax are mainly described in terms of the diagnosis, treatment, and prevention of the disease, or in the form of case reports. At the same time, some issues are less urgent from a practical point of view, such as the epidemiology of anthrax in natural ecosystems and its features in animals that are not its main hosts, while some of its non-obvious features in the main hosts remain poorly studied and have limited coverage in the scientific literature.

In this paper, we review the literature describing such aspects of anthrax. Overall, we focus on some epidemiological and clinical features of anthrax in herbivorous and carnivorous mammals, along with several other taxonomic groups of animals.

## 2. Anthrax in Ungulates

As noted above, anthrax is primarily a disease of herbivorous ungulate mammals. Cases of this disease have been described among a large number of herbivorous species, both wild and domestic [8,9,10]. Herbivores are very sensitive to anthrax infection, their disease is acute, and after a short incubation period, it leads to rapid death, sometimes without any symptoms before the terminal stage of infection [8]. The exception is pigs, which are relatively resistant to anthrax [11,12]. However, they are also not herbivores. Despite the fact that pigs are artiodactyls and are phylogenetically closely related to anthrax-sensitive animals, they are more likely to be omnivores, and differ from ungulate herbivores in the structure and physiology of their digestive system. This feature, as we discuss below, may play a key role in anthrax resistance.

Anthrax has been studied in most detail in ruminants (cattle, small ruminants, and closely related wild species, such as antelopes and deer) and equines (horses, donkeys, and zebras). This is due to a number of reasons: (1) Some of these animals form the basis of agriculture and certain industries, and in some regions, they play a role in transportation and are traditional objects of hunting. Thus, their disease has not only a noticeable economic effect, but also a high chance of being transmitted to humans. (2) Some of them, such as antelopes, zebras, rhinoceroses, bison, etc., are the basis of ecosystems preserved in wildlife sanctuaries; therefore, cases of mass disease attract increased attention. (3) Most of them are kept in large herds or live in nature in large groups, which creates conditions for the transformation of single cases into large epidemics. (4) They have large body sizes, which increases the chance of exposed corpses during epidemics, even among wild animals.

We will not dwell here on the points that are described in most studies on anthrax in herbivores, but we will describe several very remarkable epidemiological patterns, which, nevertheless, often remain out of sight.

The first point of attention concerns the “geographical features” of susceptibility to anthrax among various herbivore species. The fact is that, although almost all species are considered highly susceptible to this disease, the relative frequency of anthrax cases in different species of ungulates in each specific focus of the disease can vary significantly and be characteristic of this focus. For example, when comparing two anthrax-endemic South African ecosystems, Etosha National Park (Namibia) and Kruger National Park (Republic of South Africa), which are home to large populations of zebras and kudu, it was noted that, in Etosha Park, almost half of all the reported anthrax cases occur in zebras, while the share occurring in kudu is less than 1%. In Kruger Park, a diametrically opposite picture was observed, with more than half of the cases recorded in kudu, while zebras became sick far more rarely [8,13,14]. Such phenomena are extremely difficult to explain because we face the main problem of studying infections (including anthrax) in natural ecosystems, No matter what hypothesis we put forward, it is almost impossible to test it experimentally. It is only possible to analyze the results of field observations, which often require too much time and too much funding. Nevertheless, such hypotheses should be proposed, at least in order to imagine what exactly to confirm or refute in the future. Returning to the zebras and kudu in Namibia and South Africa, we propose two hypotheses that explain the above differences in susceptibility to anthrax. The first of them suggests that this difference in susceptibility is only apparent and that it is only a consequence of a coincidence. In this case, the difference in the incidence of anthrax does not reflect the susceptibility of animals to infection but their chances of being infected, which depend, for example, on the presence of active foci of anthrax along the routes of seasonal migrations and preferred breeding grounds for zebras and kudu in the Etosha and Kruger Parks.

The second hypothesis is less “traditional” and much more speculative. It is known that the anthrax microbe, during its short evolutionary history, became divided into three main evolutionary lineages—A, B, and C—and several canSNP groups within these lineages, and the distribution of these lineages of canSNP groups has some (not always strict) geographical patterns [15,16,17,18,19]. Etosha Park and Kruger Park differ in terms of the biodiversity of the circulating *B. anthracis* strains. In southern Africa, and in the Kruger Park directly, strains of the B lineage that are not found in Namibia circulate [20,21]. It can be assumed that these strains are capable of infecting different ungulates that differ evolutionarily and physiologically with greater efficiency than strains of line A, whether they are artiodactyl ruminant bovids (kudu) or non-artiodactyl monogastric equines (zebras). As one of the reasons for such a potential difference in virulence, we suggest differences in the sequence of anthrax toxins in strains A and B, as shown in [22].

The second in order, but perhaps the most interesting feature of anthrax in ungulates, is the fact that susceptibility to anthrax varies within different age and gender groups of the same species. While adult animals are susceptible to infection, young animals are relatively resistant, and among adults, fertile males are the most affected. This phenomenon has been described for livestock [23,24], wild bison [25,26], moose [25,27], and many African herbivores, including not only ungulates, but also Proboscidea (African elephant) [13]. There is no exhaustive explanation of these phenomena, or at least one confirmed by experiments or field observations; nevertheless, it is possible to put forward some hypotheses.

Considering that anthrax spreads in nature predominantly through the alimentary pathway, the reasons for the differences in the incidence for different age and sex groups of herbivores can be sought in the physiology of the digestive system and the feeding behavior of these groups.

**Figure 1:**
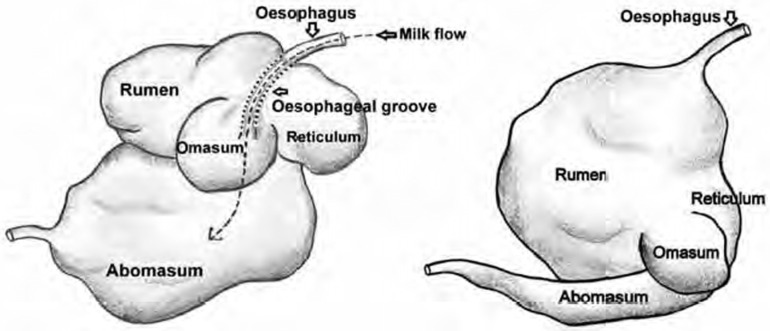
Comparative size of stomach compartments of a pre-ruminant showing oesophageal groove and of an adult ruminant age [33].

Here, it is worth making a small digression and noting that the mechanism by which a spore that has entered the gastrointestinal tract overcomes the intestinal epithelial barrier and initiates the infectious process is still not entirely clear. The following were proposed as mechanisms: (1) dendritic-cell-mediated spore invasion into intestinal Peyer’s patches [28,29]; (2) the entry of the pathogen into microtraumas caused by solid inclusions in food, such as grains of sand, fragments of lignified plant parts, bone fragments, etc. [8,23]; (3) the germination of spores in the lumen of the gastrointestinal tract, the subsequent synthesis of lysins that damage the intestinal epithelium, and the penetration of vegetative encapsulated cells through these damaged areas, which leads to the rapid development of systemic infection [30,31]. It is the latter mechanism that we can use to explain the phenomenon described above. When analyzing the reasons for the low incidence of anthrax in young animals, we can draw attention to the fact that the young feed on milk but not on plants, like adults. This, in itself, reduces the risk of infection with *B. anthracis* spores that may be present in the soil. In addition, their gastrointestinal tract is adapted for a dairy diet. Unlike adults, herbivore pups have a smaller volume and length of the gastrointestinal tract; in young ruminants, the rumen is generally underdeveloped (see Figure 1 and Table 1), and the abomasum and small intestine are mainly involved in digestion [32].

Youngsters have a lower pH in the stomach, relatively high activity of their own digestive enzymes, and a higher rate of digestion compared to adults. As a result, in terms of the structure and physiology of the gastrointestinal tract, young herbivores are, to some extent, more similar to predators than to adult herbivores. When *B. anthracis* spores are swallowed with soil at the beginning of the transition to plant food or with milk, in cases when the mother’s illness is accompanied by milk bacteremia [34], the following may apply: (1) the time spent by the spores in the gastrointestinal tract is insufficient for the germination of the spores; (2) its environment is too aggressive for the survival of vegetative cells and germinating spores. The same mechanism could explain the decreased susceptibility of predators to alimentary anthrax. We discussed this hypothesis in more detail in [35].

The shift in anthrax mortality among adult animals toward fertile males appears no less interesting. This difference in susceptibility according to age and gender groups could be due to many factors, from dietary habits to hormonal and behavioral differences. For example, food segregation between males and females of the same species may play a role: differences in the composition of the food consumed and in preferred grazing areas [25]. Males of many ungulates are larger than females and, accordingly, eat more food; thus, they are more at risk of nutritional infection. In addition, they feed on coarser vegetation. This increases both the likelihood of receiving microtraumas of the upper parts of the digestive system, which become the entrance gate of infection, and the duration of digestion, primarily in the rumen [25,27,36]. Considering the abovementioned model of spore germination as the initial stage of gastrointestinal anthrax development, such food segregation may increase the likelihood of males becoming sick when feeding on vegetation contaminated with *B. anthracis* spores. Food segregation leads to partial territorial segregation—males graze somewhat separately. This, together with the hierarchical and territorial behavior of ungulate males, suggests another mechanism that can lead to their increased incidence of anthrax. When a male dies, his territory, including preferred grazing areas, is quickly occupied by one of the competitors. If this death occurred due to anthrax, the male who reoccupied this territory is at an increased risk of infection, since it begins to graze in an epidemically dangerous area. In addition, the relatively large body size and higher aggressiveness of males means that large herbivore males avoid the risk of meeting predators to a lesser extent than females and calves [25,27]. In particular, they will be less likely to be scared away by predators gnawing the carcass of an animal killed by anthrax and contaminating the surrounding soil and vegetation, which, again, increases the risk of infection. Physiological differences, such as hormonal status, play a role in the susceptibility to anthrax of different age–sex groups. It was shown, though only in a mouse model, that the administration of different hormones changed the susceptibility to anthrax [37]. The mechanisms leading to such a reaction have not been studied in detail. In particular, it was shown that LT (lethal toxin, one of the two *B. anthracis* toxins that are major virulence factors) is a noncompetitive inhibitor of glucocorticoid-receptor activation, and the experimentally induced changing of the glucocorticoid level leads to a change in susceptibility to LT [38,39]. Although there is insufficient evidence to extrapolate from mouse models to large animal data, it can be assumed that the elevated levels of corticosteroids seen in dominant adult male ungulates [40] may also increase their susceptibility to anthrax.

The next interesting point concerns the lethality of anthrax. It is widely believed that this disease is absolutely lethal in herbivores. Nevertheless, a number of immunological studies have shown that, in nature, anti-anthrax toxin antibodies are detected in many of these animals. Therefore, they suffered anthrax that did not lead to death. As with the incidence rate, which differs from species to species within the same anthrax-endemic region, the percentage of seropositive individuals among different ungulate herbivore species living in the same region can differ significantly. For example, among buffaloes in the Serengeti (Tanzania), the number of seropositive individuals exceeded 40%; among wildebeest, it reached 20%, and no seropositive zebras were found at all [41]. However, the authors noted that, in this region, zebras and bovids (buffalo and wildebeest) differed too insignificantly in the number of cases of anthrax, so the difference in the percentage of seropositive zebras could not be explained by their low incidence of anthrax. In an article published several years later, Cizauskas et al. explained the absence of seropositive zebras in [42] by errors in the experimental methodology. In their own study in Etosha National Park, Namibia, anti-anthrax toxin antibodies were found in 52–87% of zebras, 0–15% of springboks (*Antidorcas marsupialis*), and 3–52% of elephants (the range of data is given depending on the methodology used). Thus, zebras, like other herbivores, can contract anthrax in a non-lethal form and develop a pool of protective antibodies. Interestingly, Cizauskas et al. managed to study the same individuals over several seasons. As a result, it was found that the titer of anti-anthrax toxin antibodies in zebras decreased within six months. Thus, animals could become infected again after six months, and it is likely that repeated diseases can form a more pronounced and persistent immunity. This assumption was confirmed experimentally in [43], in which serological studies of zebras after vaccination against anthrax showed that double immunization with an interval of approximately eight weeks led to the formation of reliably detectable antibodies, but the titers of these antibodies decreased to critical values after a year. Thus, the formation of long-term immunity most likely requires annual revaccination in wild ungulates. In this regard, they do not differ from domesticated animals, in which the effect of vaccination lasts no more than one year [44]. Thus, serological studies in the field show that, even in animals traditionally considered susceptible to anthrax, this disease is not always lethal. It can pass in a subacute form, and the animal can become sick with it in nature several times throughout its life.

It remains unclear exactly how this non-lethal anthrax occurs, whether we are talking about an individual reaction that allows some individuals to survive the infection, or about another route of infection in which the disease takes a mild form. It can be assumed that the bites of blood-sucking insects can be such a non-lethal route of infection. It was found that blood-sucking *Diptera*, primarily flies of the families Tabanidae and Hippoboscidae (horseflies and louse flies or keds), biting an anthrax-infected animal with bacteremia in the blood, can preserve viable *B. anthracis* cells on their oral apparatus for more than a week and in the gastrointestinal tract for more than two weeks [45]. During this period, while biting a healthy animal, they can infect it with anthrax. Experiments using blood-sucking flies on the anthrax infection of large animals have shown that a lethal infection requires at least several dozen fly bites from flies that had previously fed on a sick animal with anthrax bacteremia. Single bites do not lead to infection, or the infection passes in a relatively mild skin form and does not lead to death [46]. Such a pathway for the spread of non-lethal anthrax could well function in the wild. In this case, the role of blood-sucking flies can be twofold. On the one hand, flies can spread the infection tens of kilometers from the primary focus, and with a high population density of flies, they are able to turn isolated cases of anthrax into epizootics among ungulates [4]. On the other hand, blood-sucking flies could, in a sense, stop these epizootics, contributing to the formation of collective anti-anthrax immunity among animals susceptible to infection and, thus, maintaining the ecological balance in ecosystems endemic for anthrax.

At the end of this section, we would like to take a closer look at some of the features of anthrax in pigs. As we mentioned above, suids are considerably less susceptible to anthrax than other ungulates. At first glance, they have an increased chance to become infected with anthrax. Pigs not only graze like other ungulates, but routinely root in soils for food. Such behavior increases the chance of becoming infected if the soil is contaminated with *B. anthracis* spores. Moreover, swine are known to be opportunistic omnivores that occasionally scavenge carcasses and even prey on some livestock [47]. Consequently, pigs can consume infected animals or animals that died of anthrax, which should further increase their chances of getting infected. Nevertheless, pigs rarely get anthrax, and if they do, the clinical symptoms are limited to edema and necrotic lesions of the oropharynx and soft tissues of the head and neck [8]. Thus, the clinical manifestations of anthrax in pigs are similar to those in carnivorous mammals. We believe that the reason for this similarity is that, in terms of the structure and physiology of the gastrointestinal tract, pigs are more comparable to carnivores than to herbivores. Due to this, microtraumas of the oropharynx caused by hard food fragments apparently are the main entrance gate for anthrax infection in pigs. We could confirm this assumption by the increased mortality from anthrax in hippopotamuses, which also gnaw on corpses, including during anthrax outbreaks [48], but have a gastrointestinal tract more similar to classical herbivores, and hence, like herbivores, are susceptible to gastrointestinal anthrax. It is also worth noting that pigs are quite resistant to experimental anthrax infection, but in this indicator, too, they are quite comparable with predators (dogs) [8]. Regarding anthrax in feral pigs, we do not know of many studies on this topic. In our opinion, rather interesting results are reported in [47]. The authors found anti-anthrax toxin antibodies in a significant part of the population of Feral Swine (*Sus scrofa*) in Texas. However, most interestingly, female and adult swine tended to have higher seropositivity than male and subadult (2 months-1 year old) swine, although the measures were not statistically significant. This imbalance might be because of the social structure of wild boar, in which solitary adult males live isolated from the herd, and females live in groups of females with young boars, with four individuals representing the most frequent group size. Males aggregate to females only during the rut period, constituting groups that are more numerous [49] Therefore, either a group of females or one single male could make contact with one found source of anthrax (a corpse or a sick animal). The potential age–class bias observed could be explained by intra-group competition for such a valuable food resource as meat, with dominant adult females having an advantage over adolescents in this competition. Therefore, they have a greater chance of coming into contact with animals infected with anthrax, and a greater chance of suffering non-lethal anthrax, leading to the formation of antibodies.

## 3. Anthrax in Predatory Mammals

Predators are considered very resistant to anthrax [8,50]. The reason for their greater resistance to infection compared to ungulates remains not entirely clear. Here we can mention one interesting phenomenon described in the literature. An inverse relationship was noted between resistance to infection and sensitivity to the anthrax toxin [8,51]. However, unfortunately, this pattern was confirmed and studied in great detail (including the genetic mechanisms of this phenomenon) only for laboratory rodents, primarily for various inbred strains of mice [52]. To what extent this pattern can be confirmed for a wide range of wild predators and herbivores, and, if so, what exactly causes it in them, remains unclear at the moment. Anyway, because of this resistance, they facilitate the completion of the *B. anthracis* life cycle. By eating animals sick with anthrax, predators ensure that the vegetative cells of the anthrax microbe enter the environment, where they form spores that can cause a new cycle of herbivore infection. Anthrax resistance in predators is not absolute. It manifests itself not as an inability to become infected, but rather as an ability to transfer the disease in a non-lethal or even asymptomatic form. Anthrax has been reported in numerous species belonging to different families of predators, but their disease is often mild and the clinical symptoms are limited to edema and necrotic lesions of the oropharynx and soft tissues of the head and neck [49]. The predominance of the oropharyngeal form in predators indicates that, when eating carcasses contaminated with *B. anthracis*, microtraumas of the oropharynx, caused by hard food fragments such as bone fragments, etc., serve as the entrance gate for infection. In the lower gastrointestinal tract, *B. anthracis* vegetative cells do not survive. This is indicated by the fact that only *B. anthracis* spores are found in the feces of predators and scavengers eating animals with anthrax, and not vegetative cells [49]. This indirectly confirms the abovementioned model of alimentary anthrax, according to which the germination of spores occurs in the rumen at about neutral pH, and then the vegetative encapsulated cells enter the host organism through the epithelium of the small intestine. Due to the extremely low pH in the stomach, high proteolytic activity in the gastrointestinal tract, and high digestion rate, the described mechanism cannot be realized in predators. This may be one reason for the anthrax resistance of predators. In this case, it is possible to contract anthrax by the alimentary route only through damage to the oropharynx incurred by gnawing the hard parts of a victim’s body. This, in fact, is observed in nature.

The high susceptibility of predators to anthrax infection with mild symptoms, up to the absence of noticeable clinical manifestations, has been confirmed by the data of serological studies. For example, in [41], the results of the serological monitoring of wild animals in active foci of anthrax in Tanzania were provided. Despite the fact that hundreds of cases of anthrax among wild and domestic ungulates were recorded during the observation period, the incidence among predators was limited to single cases (one cheetah and one serval). At the same time, more than 80% of the population of the studied predators, including lions, hyenas, and even domestic dogs, were seropositive, and with a pronounced age dependence. This percentage was much higher among adult animals and, in some cases, reached 100%. Thus, in the anthrax-endemic region, predators seem to be in constant contact with *B. anthracis*, eating animals with anthrax, but the lethal disease, given its extreme rarity, apparently occurs only in certain weakened individuals.

We would like to dwell, in somewhat more detail, on the issue of anthrax resistance in cheetahs, since there is an opinion that cheetahs are one of the few predators sensitive to anthrax. Although anti-anthrax toxin antibodies were found in cheetahs, the proportion of seropositive individuals was relatively small. Good K.M et al. [53] found 1 out of 16 seropositive cheetahs in the anthrax epidemic in Botswana. Such a small proportion of seropositive animals in the “risk group”, according to the authors, can be explained by two reasons: (1) because of the small size of the sample, there is a high chance that the studied cheetahs simply did not contact sick herbivores; (2) a significant role can be played by the dietary habits of cheetahs, such as the absence of carrion in their diet, including animals that died of anthrax. Indeed, the relatively small body sizes of cheetahs, their asthenic body builds, and their predominantly solitary lifestyles do not allow them to compete for carrion or easy prey (which sick animals are) with other predators such as lions, leopards, and hyenas. For this reason, the chances of cheetahs being exposed to *B. anthracis* are relatively small. In addition, it is worth noting the specific features of the immune system of cheetahs. In the course of its evolutionary history, this species has gone through a stage of critical decline in numbers, which led to a decrease in intraspecific genetic diversity, including a decrease in the variability of the MHC (major histocompatibility complex) genes and the complete loss of some of them. This led to a decreased ability to form acquired immunity and to decreased antibody production [54,55]. At the same time, cheetahs have a high level of innate immunity that compensates for the lack of protective immunity and is possibly less energy-consuming, which is important for asthenic cheetahs practicing a hunting method associated with high energy consumption [54]. Under stress, however, innate immunity decreases to a greater extent than acquired immunity, and the animal becomes more susceptible to diseases. This mechanism led to the opinion that cheetahs are more susceptible to infectious diseases than other predators, but this opinion is based only on observations of animals kept in captivity, while free-living cheetahs seem to become sick no more often than other free-living predators [54]. Contrary to this reasoning, there is evidence of anthrax-caused deaths of free-ranging cheetahs. For example, in [56] it is reported that three free-ranging cheetahs died within 24 h after feeding on a mountain zebra that tested positive for anthrax in the Namib Desert. The authors believe the cheetahs died from anthrax. However, *B. anthracis* had not been isolated from the cheetahs’ tissues and soil samples from the accident site, so the cause of death is not undeniably proven. In any case, cheetah immune-system strategy ensures their sufficient resistance to diseases in nature, but because of their reduced ability to produce antibodies, it is quite difficult to identify by serological methods which pathogens a particular individual has encountered. However, Turnbull et al. [55] studied the immune response of cheetahs in experiments. The vaccination of cheetahs with a live anthrax vaccine led to the formation of corresponding antibodies in them. After a single vaccination, antibodies were detected after 1 month, but by 2 months after vaccination, their titer dropped to zero. Subsequent revaccination (after 11 and 12 months) led to an increase in titers to high levels, which, although they decreased, then remained at a sufficient level, where they persisted for a long time [57]. However, the antibody titer does not always correlate with protection [58], and it is not possible to directly assess the protection afforded by such vaccination in an experiment based on the challenge of vaccinated animals with virulent strains of *B. anthracis* for obvious reasons. Therefore, the protective effect of such vaccination had to be assessed indirectly, by measuring the ability of cheetah serum to protect A/J mice from infection with the Sterne 34F2 strain, to which mice are sensitive. In this experiment, the antibody titer generally correlated with the protective sera of vaccinated cheetahs (as well as vaccinated black rhinoceroses and naturally seropositive lions).

Using the example of cheetahs, we mentioned that an increase in the susceptibility of predators to infectious diseases, including anthrax, can manifest itself when they are kept in captivity. It was in zoos and on fur farms that all the known large anthrax outbreaks were recorded in predators [59,60,61]. The largest outbreaks were described for minks (*Mustela vison*) kept on fur farms. Such cases are very interesting, since a large number of individuals (usually at least several hundred) were present on the farm at the same time, under constant supervision, and all these animals were centrally and simultaneously exposed to the risk of infection from one source, which, in all the cases described, was infected feed. All this taken together provided an excellent opportunity to observe epidemic patterns. The clinical picture in diseased minks was the same: a period of the enlargement of the spleen, bacteremia in the blood, and edema in the lungs [59,62]. It is interesting that not all of the minks that received contaminated food fell ill; on average, about a quarter fell ill (according to the average results of several outbreaks), and during some outbreaks, only isolated cases of the disease were recorded. For example, in one of the described cases, only two females of the total number of minks kept on the farm fell ill. Previously, they received bites from males during the mating process. Therefore, the cause of the disease could well be a violation of the skin with the possible penetration of the pathogen from the teeth of the males (who had previously eaten infected meat) directly into the blood. It is also possible that hormonal changes during mating increased susceptibility to alimentary anthrax infection. Unfortunately, during outbreaks, no studies of the causes of such a selective incidence were carried out, and during the outbreaks in minks, several cases of anthrax were reported in other animals kept on the same farms and fed the same contaminated food as the infected minks. Only fur-bearing animals contained in cages, such as foxes, raccoons, and badgers, became ill, while cases of anthrax in domestic dogs and cats were not found, despite the presence of anthrax-infected meat in their diet [59]. However, we do not believe that there is any reason to believe that fur-bearing animals are more susceptible to anthrax than domesticated predators. Keeping fur animals in captivity results in high population density, limited movement, and constant contact with people, which leads to permanent stress. This stress, as mentioned above, can be the cause of reduced immunity and increased susceptibility of predators to infectious diseases, including anthrax. Domesticated carnivores, such as dogs and cats, do not experience such stress, as they are kept in the best conditions and constant human contact is natural for them. As a result, they show a natural resistance to anthrax that is not reduced by stress.

Here, we would like to note that the resistance of carnivores to anthrax is illustrated not only by the rarity of cases of the disease [63] and a high percentage of seropositive individuals in active natural anthrax foci [41], but also by low virulence in experiments. According to data provided in [8], the LD_50_ values for dogs were 5 × 10^10^ for parenteral infection and 1.8 × 10^7^ for inhalation infection. Attempts to induce experimental pulmonary anthrax in dogs using lower doses (1–4 × 10^5^) made by Gleiser et al. [11] were ineffective. The only visible result was an increase in body temperature and fever in some individuals. *B. anthracis* was found in all individuals until the 30th day in the lungs (at the injection site) but was not detected in the blood and spleen. In other words, the infection did not develop.

Such data give the impression that predators are especially resistant to experimental anthrax, since the LD_50_ values for dogs given in [8] were higher than the LD_50_ values for some other animals given there. At the same time, where [52] is cited in [8], it was noted that cattle are also extremely resistant to parenteral infection with *B. anthracis*. Thus, it can hardly be unequivocally stated that there is a difference between carnivores and herbivores in terms of resistance to anthrax when infected by injection, such that *B. anthracis* directly enters into the internal environment of the host organism. If there is such a difference, then it would not be possible to detect it, since it requires experiments that are unlikely to be carried out in the foreseeable future, for both ethical and economic reasons.

## 4. Anthrax in Rodents

As we have mentioned above, anthrax mainly affects ungulates and can affect humans. Therefore, the greatest practical interest is in the study of anthrax in ungulates and humans. Modeling anthrax on ungulates and human-mimicking primates, however, is unreasonably expensive and difficult. Therefore, researchers are forced to use small rodents (mice, guinea pigs, and rats) and rabbits as experimental animals for anthrax studies. This is the main problem in the study of anthrax—the necessity of studying the alimentary disease of hoofed herbivores through the injection infection of small rodents. Even when modeling not gastrointestinal but pulmonary or skin anthrax, it is not always possible to extrapolate the data obtained in experiments to large animals. We will not dwell here on the features of using laboratory rodents and rabbits for anthrax experiments; these issues are covered in great detail in other articles. For example, we can recommend an excellent review written by Welkos [52].

In this paper, we would like to briefly discuss rodent anthrax in nature. Despite the fact that many data on experimental anthrax in mice, rats, and guinea pigs have accumulated, the epidemiology of anthrax in wild rodents is rarely studied. We found only one study on experimental anthrax susceptibility in several wild rodent species [64]. Of the 12 species studied, most were susceptible to intradermal infection, and the DCL was 80 spores of the virulent strain. Two species of kangaroo rats (*Dipodomys ordii* and *Dipodomys microps*) were resistant to infection, the LD_50_ ranging from 20,000 to 40,000 spores, and grasshopper mice (*Onychomys leucogaster*) and cliff chipmunks (*Eutamias dorsalis*) showed significant individual differences in their response to infection that did not allow the making of an unambiguous conclusion about the species’ susceptibility to anthrax. We also found mention that black rats (*Rattus rattus*) showed high resistance to infection—non-germinated spores of *B. anthracis* were found in their blood 30 days after the intravenous administration of 10^7^ spores, without visible symptoms [12]. At the same time, rats, at least laboratory ones, are extremely sensitive to injections of anthrax toxin, in contrast to laboratory mice, which are more resistant to the toxin, but very susceptible to anthrax infection [52]. Unfortunately, we do not know whether this pattern is true for wild mice—voles, house mice and other species.

In general, despite the lack of data and significant fluctuations in species susceptibility, rodents are likely to be resistant to infection in natural conditions. The main argument, which indirectly but clearly indicates this, is that anthrax outbreaks in herbivores are not accompanied by disease and the mass mortality of wild and synanthropic rodents, despite the rodents having access to the same food (which is the source of infection in most cases) as the diseased animals. Moreover, rodents, if they have the opportunity, eat the corpses of dead animals [65], which can also be a source of infection. This observation fits with the hypothesis we mentioned, where a key role in the differential susceptibility to anthrax in natural conditions is played by anatomical differences in the gastrointestinal tract and the physiology of digestion. The omnivorous nature of rodents and the extremely high rate of their digestion (due to their small body size and, consequently, high metabolic rate) cause *B. anthracis* vegetative cells to die in the gastrointestinal tract, and the spores do not have enough time for germination. This was confirmed by the results of the experiments described in [30]. In this work, in order to make mice susceptible to gastrointestinal anthrax, the authors reduced the activity of their gastrointestinal tracts by prolonged fasting and chemically neutralizing the pH in the stomach.

## 5. Anthrax in Birds

Although individual cases, and even epizootics, of anthrax in birds have been mentioned in the literature [8,66], these references, in most cases, do not contain detailed descriptions of the epidemic picture, pathogenesis features, and references.

There are reported cases of anthrax in various bird species—eagles, vultures, cranes, and domestic ducks and geese. The disease is manifested by edema in the neck; hemorrhagic enteritis; hemorrhages on the surfaces of internal organs; the swelling of the kidneys, spleen, and liver; and the accumulation of fluid in the chest and abdominal cavities, but it can also be mild, and limited to the appearance of carbuncles on the ridges and limbs [64]. In general, there are very few such reports; therefore, anthrax in birds is extremely rare. Perhaps one of the reasons for this is the high body temperature of birds (which varies among species but often exceeds 40 °C), at which *B. anthracis* cannot grow normally. This hypothesis was confirmed by the relatively high susceptibility to anthrax in ostriches, which have a relatively low body temperature (38–39 °C). They are susceptible to anthrax, and mass cases of the disease have been repeatedly described, both in the wild and on farms [41,67,68,69]. This frequency of anthrax cases has made it possible to describe the clinical picture in detail. In ostriches, anthrax can occur in two clinical forms: (1) sudden death without a preliminary increase in the severity of symptoms, where bacteremia in the blood, petechial hemorrhages in the pleura and peritoneum, and sometimes enlargement and darkening of the spleen are detected only posthumously; (2) fever, accompanied by drowsiness, anorexia, and a generally depressed state. This form of the disease often ends in spontaneous recovery and is easily amenable to antibiotic therapy. Both forms can occur simultaneously in different individuals in the same flock during the same anthrax outbreak. Therefore, they are more likely to be individual reactions of birds to infection rather than features of the *B. anthracis* strain causing a particular outbreak. Despite being more susceptible to anthrax than other birds, ostriches seem to be more resistant to this infection than ungulates. This has been evidenced by cases of anthrax outbreaks among cows caused by feed contaminated with *B. anthracis*, during which no cases of anthrax were recorded among the ostriches kept on the same farms and fed the same contaminated feed [69].

Again, we take the liberty of returning to the “digestive” hypothesis to explain the reduced susceptibility of birds to anthrax. The use of flight means a very energy-intensive mode of locomotion, a relatively small body weight, and consequently, a high speed and activity of digestion, which is necessary for both ensuring such a fast metabolism and reducing body weight (by reducing the amount of food digested at a particular time in the gastrointestinal tract). These factors, along with high body temperature, may prevent the possibility of gastrointestinal anthrax infection. Both the “digestive” and “temperature” hypotheses together, complementing each other, can explain the relative susceptibility of ostriches to anthrax. Ostriches are very large and do not fly, so they have a less active metabolism, which leads to both a decrease in the speed of digestion (which may be the reason for a slightly higher likelihood of food infection) and a decrease in body temperature down to the values at which *B. anthracis* is able to exist.

While discussing the possibility of anthrax infection in birds, we would also like to mention the work [70] which reports the discovery of antibodies to the anthrax toxin in vultures. The authors question whether the presence of these antibodies means infection per se in vultures or absorption of incompletely digested epitopes of the toxin or both. This is quite an interesting thought. The idea of anti-anthrax toxin antibodies production as a response to the entry of anthrax toxin into the digestive tract without anthrax infection is somewhat speculative in our opinion. However, it could be assumed that the same mechanism of forming an immune response without disease can work in other predators and scavengers.

It is worth dwelling separately on the epidemiological aspect of bird resistance to anthrax infection. Immunity to infection, coupled with the high mobility afforded by the ability to fly, make scavenger birds an ideal vector for anthrax. After pecking the corpse of an anthrax-infected animal, birds are able to carry *B. anthracis* vegetative cells in pieces of the corpse or on their bodies (or carry the spores in excrement) over distances of tens of kilometers. What is especially important is that birds in this process can overcome geographic barriers that are insurmountable for terrestrial animals—rivers, straits, mountains, human settlements, etc. To support our arguments, we can refer to articles that have considered the significant roles of vultures in southern Africa [71], and gulls and crows in northern Canada [72], in the spread of anthrax, and vultures and some facultative avian scavengers in the United States [73].

## 6. Anthrax in Cold-Blooded Vertebrates

Fish, amphibians, and reptiles are apparently resistant to anthrax; natural cases of the disease in these animals have not been described. Experimental anthrax has been induced in amphibians and reptiles, but it has required an artificial increasing of their body temperatures [66]. Thus, if birds’ body temperatures are too high, in reptiles and amphibians, on the contrary, they are too low for the existence of an anthrax microbe. It is very doubtful that an increase in body temperature for a period that allows infection to develop can occur in natural conditions. However, there are some case reports in which the possibility of anthrax infection in reptiles was suspected. All such cases were associated with unexpected or excessive deaths of reptiles during anthrax outbreaks in susceptible animals, but studies to determine the cause of these deaths and whether they were due to anthrax infection have not been conducted [8]. For example, overeating caused by access to an abnormally large number of carcasses of dead hippopotami has been cited as a possible cause of extra-mortality of crocodiles during an anthrax outbreak in hippopotami in Zambia in 1988–1989 [8].

At the end of this short section, we would like to say a few words about anthrax in fish. Like other cold-blooded animals, fish do not suffer from anthrax, but in some cases, they can be a vector of this infection. According to some reports, *B. anthracis* can persist in algae, including those on the bodies of fish [74]. In addition, fish can eat the corpses of animals that died from anthrax when they enter water. Cutting such fish and eating them without proper heat treatment can lead to anthrax infection [8].

In the end, the absence of confirmed cases of anthrax in cold-blooded vertebrates in nature, or reported reliable cases of their participation in the spread of anthrax infection, allows us to consider their epidemic significance as zero.

## 7. Conclusions

Summing up the general epidemic patterns of the spread of anthrax in nature, we observe that different taxonomic, non-taxonomic, and even intraspecific groups of animals show differences in susceptibility to infection and the severity of the infectious process. It could be assumed that this picture was formed under the influence of evolutionary selection for susceptibility to anthrax, which affects different groups of macro-organisms with different efficiency. Despite the fact that *B. anthracis* is quite widespread in the modern world, this species, according to currently accepted ideas, arose and spread relatively recently, and its history covers no more than a few tens of millennia [15]. In addition, there is reason to believe that such a wide distribution has an anthropogenic cause and arose quite recently [21,35]. Most of the taxa of modern mammals were formed in terms estimated as millions of years ago [75,76], and they are incomparably older than *B. anthracis*. In particular, the main strategies of the immune system and the structural and regulatory elements characteristic of a particular taxon—species, genus, family, and so on—evolved much earlier than its first encounter with the anthrax microbe. Therefore, most likely, the difference in susceptibility to anthrax is not a consequence of the adaptation of certain groups of animals to the effects of *B. anthracis* but of the mechanisms of pathogenicity of the anthrax microbe itself, evolutionarily developed to overcome the immune response of the host macro-organism. These features of the interaction between macro- and micro-organism, expressed in the selective virulence and particulars of the infectious process, are yet to be adequately studied for a more accurate prediction of the epidemic situation in natural and anthropogenic ecosystems and for the more accurate modeling of anthrax infection in laboratories.

## Figures and Tables

**Table 1:** Proportion (as percent) of total tissue weight or volume contributed by different compartments of pre-ruminant stomach as influenced by age [33].

	Age in Weeks						
	0	4	8	12	16	20–26	34–48
Cattle							
Reticulo-rumen	38	52	60	64	67	64	64
Omasum	13	12	13	14	18	22	25
Abomasum	49	36	27	22	15	14	11
Buffalo							
Reticulo-rumen	69	88	93			95	
Omasum	2	2	1			4	
Abomasum	29	10	6			1	
Sheep							
Reticulo-rumen	8–10					70–80	
Omasum	15–20					15–20	
Abomasum	70–80					8–10	

## Data Availability

Not applicable.
